# Activated mast cells in periprosthetic joint infection-associated tissue

**DOI:** 10.3389/fimmu.2023.1183977

**Published:** 2023-08-16

**Authors:** Cody R. Fisher, Robin Patel

**Affiliations:** ^1^ Mayo Clinic Graduate School of Biomedical Sciences, Department of Immunology, Mayo Clinic, Rochester, MN, United States; ^2^ Division of Clinical Microbiology, Department of Laboratory Medicine and Pathology, Mayo Clinic, Rochester, MN, United States; ^3^ Division of Public Health, Infectious Diseases, and Occupational Medicine, Department of Medicine, Mayo Clinic, Rochester, MN, United States

**Keywords:** mast cells, periprosthetic joint infection, non-infectious arthroplasty failure, periprosthetic tissues, tryptase

## Abstract

**Background:**

Periprosthetic joint infection (PJI) is a devastating complication of total joint arthroplasty surgery. Increased densities of activated mast cells have been predicted to be present in PJI compared to non-infectious arthroplasty failure based on analysis of transcriptomic data, but their presence in PJI-associated periprosthetic tissues has not been visually confirmed.

**Objective:**

This preliminary study investigated the presence and activation status of mast cells in periprosthetic tissues associated with PJI.

**Methods:**

Periprosthetic tissues from five PJI cases and three arthroplasty failures due to instability and one due to stiffness were immunohistochemically stained using tryptase and microscopically evaluated to enumerate mast cells and evaluate overall activation status within tissue samples. Mast cell activation was evidenced by the release of tryptase into the extracellular space surrounding mast cells.

**Results:**

Mast cells were found in all samples, with average cellular densities of 22 and 26 cells/mm^2^ tissue in PJI and uninfected samples, respectively (p, 0.6610). Apparent mast cell activation and degranulation was readily observed throughout each of the five PJI samples studied, but not in any of the uninfected samples studied.

**Conclusion:**

While preliminary, these findings provide evidence for a role of mast cells in the immune response in PJI. Additional investigation of the role of mast cells during arthroplasty failure is warranted, providing a better understanding of underlying biology and informing potential diagnostic and treatment targets.

## 1 Introduction

Total joint arthroplasty (TJA) is a common surgical procedure to treat joint pain and restore mobility. Arthroplasty failure is a major complication of TJA, caused by periprosthetic joint infection (PJI) and several non-infectious etiologies, including instability, stiffness, and aseptic loosening (1, 2). PJI is associated with biofilm formation by bacteria growing on implanted devices. Arthroplasty failure may necessitate surgical intervention, with intensive antimicrobial treatment also needed for PJI.

Recently, our group used an RNA-sequencing-based cellular deconvolution method, CIBERSORTx, to predict immune cellularity based on interrogation of bulk transcriptomic data from PJI and non-infectious arthroplasty failure (NIAF) sonicate fluid (derived from sonicating resected implants) (3). This evaluation suggested that activated mast cells may be elevated in PJI compared to NIAF, based on an elevation of a predicted activated mast cell transcriptomic signature informed by a combination of IL1A, IL1B, CCL4, and CCL20 expression. While these results gave evidence of a potential role of mast cells in the immune response to PJI, they were limited by the analytical method, as cellular populations were based solely on predictive deconvolution algorithms and not on actual cellular analyses.

Connective tissue resident mast cells are known to act as “sentinel cells” upon stimulation by bacteria during infection, detecting pathogen-associated molecular patterns, and initiating downstream antimicrobial inflammation through secretion of extracellular tryptase and chymase proteases, recruitment of phagocytic neutrophils, and direct antigen presentation to the adaptive immune response (4–7). The presence and inflammatory roles of synovial mast cells in rheumatoid arthritis, osteoarthritis, and arthrofibrosis has been described (8–11). Additionally, the presence and activation of mast cells in tissues surrounding implanted hips have previously been shown during aseptic loosening, although their role in other causes of NIAF is unexplored (12, 13). While infiltration and inflammatory impact of other cell types, such as neutrophils and macrophages, is well-described in PJI, the potential presence and role of mast cells during PJI were a novel finding.

This preliminary study aimed to determine whether activated mast cells can be visualized in peri-prosthetic tissues during PJI, confirming results of a prediction made based on transcriptomic analysis of sonicate fluid.

## 2 Materials and methods

### 2.1 Patient sample cohort

Periprosthetic tissue samples were taken during knee and hip arthroplasty revision surgery, as approved by the Mayo Clinic Institutional Review Board (09-000808). Paired tissues from NIAF sonicate fluids (n=4) with the lowest predicted activated mast cell populations and from PJI sonicate fluids (n=5) with the highest predicted activated mast cell populations by cellular deconvolution (3) were examined and are described in **Table 1**. The average age of the subjects studied was 66.4 years and ranged from 52 to 78 years. Five of the nine (56%) were from male subjects. NIAF samples included those associated with instability (n=3) and stiffness (n=1). PJI was determined by Musculoskeletal Infection Society (MSIS) diagnostic criteria (14) and was associated with *Staphylococcus epidermidis* (n=2), *Actinomyces naeslundii* (n=1), *Pseudomonas aeruginosa* (n=1), and *Streptococcus mitis* (n=1). Three of the five PJI subjects had had symptoms lasting ≥1 year, with two having had symptoms lasting <1 year (15). One PJI sample was from a total hip arthroplasty revision surgery, with the remaining samples, including four with PJI and four with NIAF, from total knee arthroplasty revision surgeries. The average age of the implant for each sample was 4.6 years, ranging from 0.6 to 10.7 years.

**Table 1:** Sample cohort characteristics.

Type		Age (years)	Sex	Implant location	Implant age (years)	Symptom duration (years)
Periprosthetic joint infection (pathogen shown below)						
	*Actinomyces naeslundii*	60	Male	Hip	9.1	1.1
	*Pseudomonas aeruginosa*	78	Female	Knee	10.7	1.1
	*Staphylococcus epidermidis*	65	Male	Knee	0.6	0.6
	*S. epidermidis*	59	Male	Knee	2.6	1.7
	*Streptococcus mitis*	77	Female	Knee	3.7	0.2
Non-infectious arthroplasty failure (type shown below)						
	Instability	71	Female	Knee	2.4	0.3
	Instability	52	Female	Knee	2.8	1.1
	Instability	58	Male	Knee	3.1	0.1
	Stiffness	78	Male	Knee	6.0	0.2

### 2.2 Immunohistochemical staining, microscopy, and image analysis

Formalin-fixed paraffin-embedded (FFPE) tissue blocks were sectioned and stained using a clinically validated immunohistochemistry (IHC) staining protocol with anti-mast cell tryptase antibody AA1 (Agilent Dako, Santa Clara, CA, USA) at a 1/2,000 dilution (16–19). Protease 2 was used as a pretreatment step with OptiView DAB IHC Detection. IHC-stained slides were imaged using a KEYENCE BZ-X800 All-in-One Fluorescence Microscope (KEYENCE, Osaka, Japan). The microscopist was blinded as to whether samples were from PJI or NIAF. For imaging, 31 × 21 total fields at 10×, accounting for an approximately 32 mm × 16 mm slide area, were stitched together using the BZ-X800 Analyzer (KEYENCE). The numbers of mast cells and total tissue surface areas within stitched images were determined using ImageJ v1.53a (20). Mast cell activation was evaluated under 40× and 100× magnification.

### 2.3 Statistical analysis and graphical representation

Mast cells per mm^2^ tissue sample were calculated and plotted using GraphPad Prism 9 v9.5.1 (San Diego, CA, USA). Statistical significance was determined via Welch’s two-sample t-test.

## 3 Results

**Figure 1:**
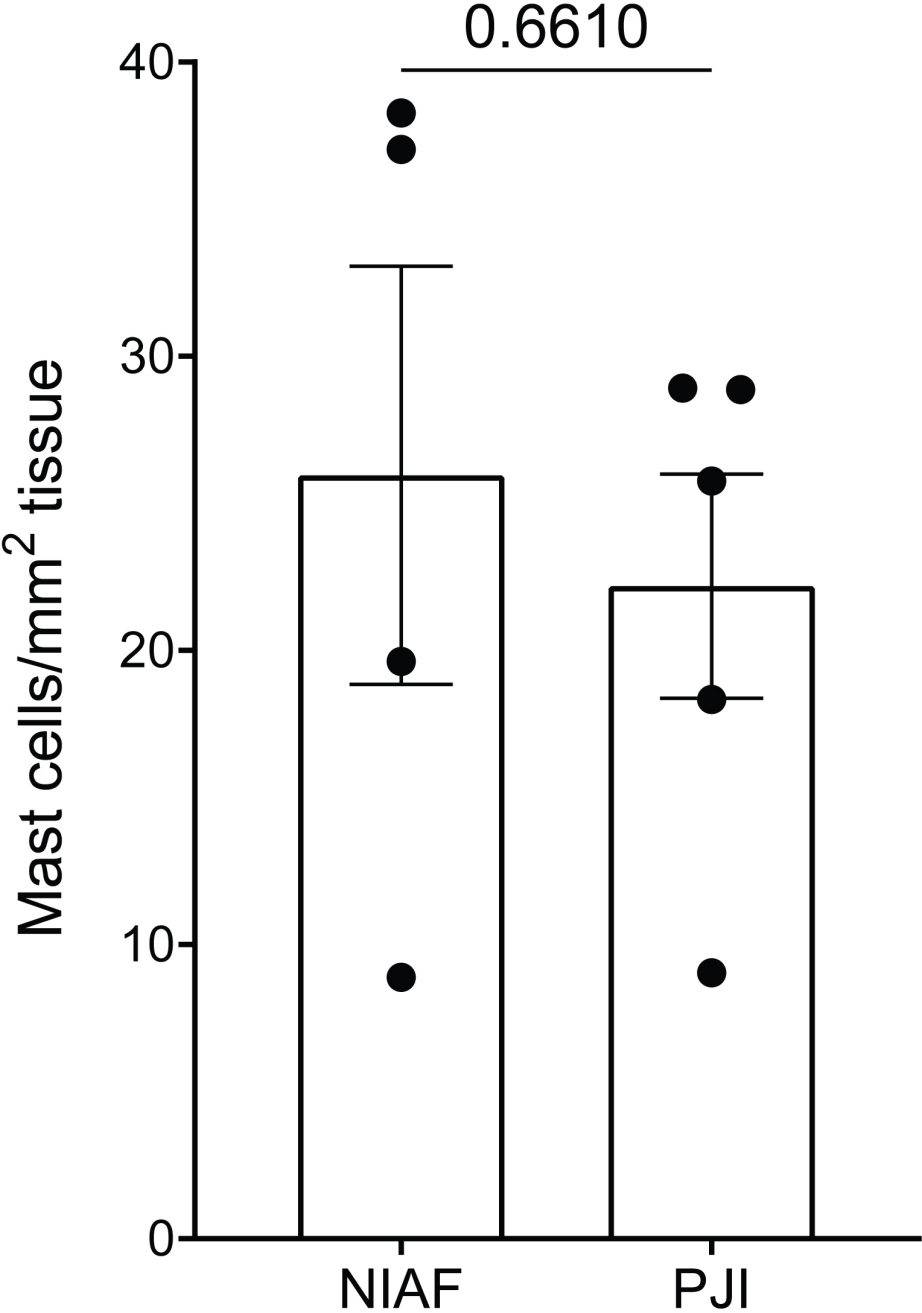
Density of mast cells in periprosthetic tissue from non-infectious arthroplasty failure [NIAF; instability (n=3) and stiffness (n=1)] and periprosthetic joint infection (PJI, n=5) samples. Total mast cells count per mm^2^ tissue in NIAF and PJI samples were enumerated by tryptase immunohistochemical staining. Statistical significance was determined via Welch’s two-sample t-test.

Tryptase IHC-stained slides were histopathologically evaluated to determine mast cell densities in periprosthetic tissues. Average mast cell densities in PJI and NIAF samples were 22 and 26 cells/mm^2^ tissue, respectively (**Figure 1**; Welch’s two-sample t-test p-value of 0.6610).

**Figure 2:**
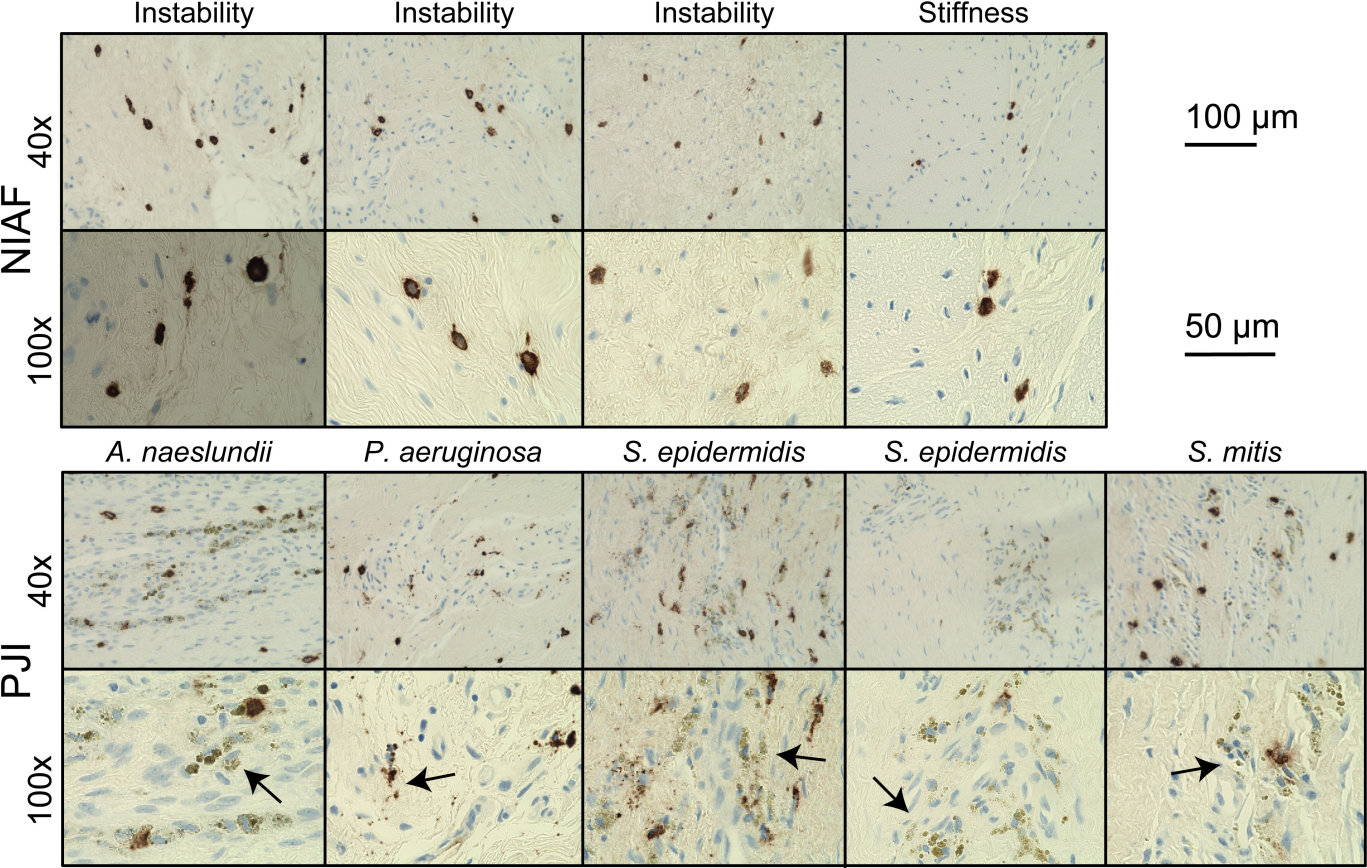
Mast cell activation in periprosthetic tissue from arthroplasty failure due to instability, arthroplasty failure due to stiffness, and periprosthetic joint infection (PJI). Representative images of mast cells in instability or stiffness (top) and PJI (bottom) samples at 40× and 100× magnification, showing apparent degranulation of activated mast cells (arrows) by tryptase immunohistochemical staining in PJI. Scale bars indicate 200 µm at 40× and 100 µm at 100×. Images depicted are from two causes of non-infectious of arthroplasty failure (NIAF), instability (n=3) and stiffness (n=1), and five PJIs, including *Actinomyces naeslundii* (n=1), *Pseudomonas aeruginosa* (n=1), *Staphylococcus epidermidis* (n=2), and *Streptococcus mitis* (n=1) PJI.

Apparent mast cell activation and degranulation were evaluated microscopically in each of the samples. A release of tryptase from mast cells into the extracellular space was observed throughout each of the PJI samples, whereas tryptase in uninfected tissues remained largely punctate, isolated within cellular membranes, and not released into the surrounding tissue (**Figure 2**).

## 4 Discussion

In this preliminary study, periprosthetic tissues from five PJI and four uninfected arthroplasty failure cases were stained for tryptase, microscopically imaged, and analyzed to determine and compare overall mast cell density and activation status. Results, although preliminary, show for the first time the presence of mast cells in PJI-associated periprosthetic tissues, supporting a potential role for mast cell activation in the immune response in PJI.

Limberg et al. recently described enumerated mast cell densities in periprosthetic tissues from patients undergoing total knee arthroplasty for arthrofibrosis, a subtype of NIAF (10). Mast cell densities found here not only recapitulate those previously reported in non-infected tissues but also show similar numbers of mast cells in PJI-associated tissues. Overall, there was no statistically significant difference in mast cell numbers between PJI and NIAF samples. This is not unexpected as mast cells are considered long-lived tissue-resident cells initially seeded from fetal origins and, therefore, not expected to infiltrate into the tissues during infection (7). Of particular interest, however, was that mast cells appeared to be activated in PJI samples, with apparent increased release of extracellular tryptase, compared to the limited number and types of NIAF samples evaluated. These results support those of previously reported RNA-based predicted activated mast cell populations in sonicate fluid samples from PJI (3).

As known contributors to the chemoattraction and activation of innate immune cells during other types of bacterial infection (4–7), the results found here provide evidence for a potential role of activated mast cells in the infiltration of macrophages and neutrophils into the synovium during PJI (2, 21). Local therapeutic targeting to activate mast cells has recently been shown to enhance clearance of bacterial skin infections in mice and limit reinfection (22). A similar approach may be relevant to the treatment of PJI, paired with traditional antimicrobial treatment.

In this study, there was a lack of presumed degranulation in instability- and stiffness-associated NIAF samples. This is in contrast with previous studies, where degranulation of mast cells was observed in hip tissues from aseptic loosening (12, 13). This may be because the non-infected tissues samples analyzed here were chosen from paired sonicate fluids having low predicted activated mast cell populations, from results of transcriptomic-based cellular deconvolution. There may be differences in mast cell activation related to the underlying pathology causing NIAF. Alterations in activation status between different etiologies of NIAF are of particular interest, as they may be a potential differentiative biomarker. As such, further investigation into mast cell activation in different types of NIAF is warranted.

This preliminary study has several limitations. First, the sample cohort examined was small and not necessarily fully representative of the disease states studied; tissues were from only four NIAF and five PJI subjects and were specifically picked based on previously predicted activated mast cell quantification by cellular deconvolution in associated sonicate fluids (3). In addition, only two etiologies of NIAF, instability and stiffness, were evaluated (12, 13). While limited, this cohort does provide initial confirmatory characterization of mast cells within periprosthetic tissues associated with PJI. Next, this study lacks inclusion of native joint tissues. Whether mast cell densities and activation status differ between tissues from joints with or without implanted devices is unknown. Lastly, while this staining method is clinically validated to determine mast cell quantification, only a single mast cell stain, tryptase IHC, was utilized to determine both cell densities and activation status. Confirmatory investigations using additional cellular quantification and activation status assessment methods are needed.

The findings presented support further investigation and characterization of the role of mast cells in PJI.

## Data availability statement

The original contributions presented in the study are included in the article/supplementary material. Further inquiries can be directed to the corresponding author.

## Ethics statement

The studies involving human participants were reviewed and approved by the Mayo Clinic Institutional Review Board (09-000808 - “Detection of Biofilms on Explanted Orthopedic Devices”). The ethics committee waived the requirement of written informed consent for participation.

## Author contributions

CF contributed to study design, conducted laboratory experiments and data analysis, and drafted the manuscript. RP contributed to study conception, study design, and drafting of the manuscript. Both authors read and approved the final manuscript.
